# Ilheus and Saint Louis encephalitis viruses elicit cross-protection against a lethal Rocio virus challenge in mice

**DOI:** 10.1371/journal.pone.0199071

**Published:** 2018-06-13

**Authors:** Alberto Anastacio Amarilla, Marcilio Jorge Fumagalli, Mario Luis Figueiredo, Djalma S. Lima-Junior, Nilton Nascimento Santos-Junior, Helda Liz Alfonso, Veronica Lippi, Amanda Cristina Trabuco, Flavio Lauretti, Vanessa Danielle Muller, David F. Colón, João P. M. Luiz, Andreas Suhrbier, Yin Xiang Setoh, Alexander A. Khromykh, Luiz Tadeu Moraes Figueiredo, Victor Hugo Aquino

**Affiliations:** 1 Laboratory of Virology, Department of Clinical Analyses, Toxicology and Food Sciences, Faculty of Pharmaceutical Sciences of Ribeirao Preto, University of Sao Paulo, Ribeirao Preto, Sao Paulo, Brazil; 2 Virology Research Center, Ribeirao Preto Medical School, University of São Paulo, Ribeirao Preto, São Paulo, Brazil; 3 Department of Biochemistry and Immunology, Ribeirao Preto Medical School, University of Sao Paulo, Ribeirao Preto, SP, Brazil; 4 Department of Neurosciences and Behavioral Sciences, Ribeirao Preto Medical School, University of São Paulo, Ribeirao Preto, Brazil; 5 Laboratory of Inflammation and Pain, Department of Immunology, Ribeirao Preto Medical School, University of São Paulo, Ribeirao Preto, Sao Paulo, Brazil; 6 Australian Infectious Diseases Research Centre, School of Chemistry and Molecular Biosciences, The University of Queensland, St Lucia, Australia; 7 QIMR Berghofer Medical Research Institute, Brisbane, Australia; Center for Disease Control and Prevention, UNITED STATES

## Abstract

Rocio virus (ROCV) was the causative agent of an unprecedented outbreak of encephalitis during the 1970s in the Vale do Ribeira, Sao Paulo State, in the Southeast region of Brazil. Surprisingly, no further cases of ROCV infection were identified after this outbreak; however, serological surveys have suggested the circulation of ROCV among humans and animals in different regions of Brazil. Cross-protective immunity among flaviviruses is well documented; consequently, immunity induced by infections with other flaviviruses endemic to Brazil could potentially be responsible for the lack of ROCV infections. Herein, we evaluated the cross-protection mediated by other flaviviruses against ROCV infection using an experimental C57BL/6 mouse model. Cross-protection against ROCV infection was observed when animals had prior exposure to Ilheus virus or Saint Louis encephalitis virus, suggesting that cross-reactive anti-flavivirus antibodies may limit ROCV disease outbreaks.

## Introduction

Rocio virus (ROCV) was the causative agent of an unprecedented outbreak of encephalitis during the 1970s in the Vale do Ribeira, Sao Paulo State, in the Southeast region of Brazil [1]. Approximately 900 cases were detected in more than 20 municipalities in the region; the case-fatality rate was 13% and neurological sequelae were observed in 20% of survivors [2, 3]. The disease was mainly characterized by an acute onset of fever and headache, in addition to clinical signs and symptoms of central nervous system (CNS) involvement such as meningeal irritation, consciousness alterations and motor impairment [4]. The ability of ROCV to inhibit alpha/beta interferon (IFN-α/β) signaling may determine viral pathogenicity [5]. Surprisingly, no further cases of ROCV infection have been reported after this outbreak; however, serological surveys have suggested that unreported infections with ROCV among humans and animals in different regions of Brazil are still occurring [6–9].

ROCV is a member of the *Flaviviridae* family, *Flavivirus* genus. Its genome consists of a single-strand, positive-sense RNA approximately 11kb in length, containing a single open reading frame (ORF) that encodes a polyprotein, which is cleaved into structural [capsid (C), pre-membrane (prM) and envelope (E)] and non-structural proteins (NS1, NS2A, NS2B, NS3, NS4A, NS4B and NS5) [10, 11]. Mosquitoes of the *Psorophora* and *Culex* genera may be involved in the natural transmission cycle of ROCV [12]. Serological characterization classified ROCV as part of the Japanese encephalitis virus (JEV) serogroup, which includes encephalitis-causing agents such as West Nile virus (WNV), Murray Valley encephalitis virus (MVEV) and Saint Louis encephalitis virus (SLEV) [1].

In Brazil, thirteen different flaviviruses have been isolated including *Yellow fever virus* (YFV), *Dengue virus* (DENV types 1–4), SLEV, *Cacipacore virus* (CPCV), *Iguape virus* (IGUV), ROCV, *Ilheus virus* (ILHV), *Bussuquara virus* (BUSQV), WNV and the more recently introduced *Zika virus* (ZIKV) [13–15]. Although DENV is still the most medically important flavivirus circulating in Brazil, serological surveys have suggested that ILHV, SLEV, BSQV, CPCV, YFV and ZIKV are also actively circulating in different regions [8, 9, 13, 16].

Flaviviruses show a close antigenic relationship that allowed the classification of these viruses by cross-neutralization tests into several antigenic subgroups or serocomplexes [17–19]. In addition, cross-protection among flaviviruses, even among members of different serocomplexes is well documented. For example, (i) infection with DENV can protect against subsequent JEV, SLEV and WNV infections [20–22], (ii) ZIKV infection confers protection against subsequent WNV infection [23] and (iii) JEV and SLEV infections can protect against WNV and DENV infections [24–27]. Consequently, cross-protective immunity induced by flaviviruses endemic to Brazil could potentially be responsible for the lack of ROCV infections. In this study, we analyzed the cross-protection in mice induced by flaviviruses circulating in Brazil against ROCV and showed that prior infection with ILHV or SLEV protected mice against lethal ROCV challenge.

## Material and methods

### Animals

Six week-old Balb/c and C57BL/6 female mice were obtained from the Central Animal Facility at the University of Sao Paulo, Ribeirao Preto, Sao Paulo State, Brazil. Blood was obtained from the retro-orbital region under ketamine/xylazine anesthesia to achieve adequate immobilization and to reduce the stress and pain of the animals. All experiments were performed in a biosafety level-3 facility at the Virology Research Center, Medical School of Ribeirao Preto, University of Sao Paulo.

### Ethics statement

All animal experiments were performed according to the guidelines of the Brazilian College of Animal Experimentation. The Ethics Committee on Animal Experimentation of the Medical School of Ribeirao Preto, University of Sao Paulo, approved this study (Permit No. 022/2015-1).

### Cells and viruses

*Aedes albopictus (*C6/36) cells were maintained in the Roswell Park Memorial Institute (RPMI) 1640 medium, while Vero cells in Dulbecco's Modified Eagle's medium. Media was supplemented with 10% fetal bovine serum (FBS), penicillin (100 U/mL) and streptomycin (100 μg/mL) (Gibco, USA). C6/36 (Cells Bank of Rio de Janeiro, BCRJ 0343) and Vero (BCRJ0245) cells were grown at 28°C and 37°C, respectively. The flaviviruses BSQV (BeAn 4073), CPCV (BR/SP/CPCV/A. CAJENNENSE/1997), DENV-1(Mochizuki), DENV-2 (NGC), DENV-3 (D3BR/SL3/02), DENV-4 (H241), ILHV (BeH 7445), SLEV (BeH35596), YFV (17DD), ZIKV (ZKV001) and ROCV (SPH34675) were used in this study. All virus stocks were generated using C6/36 cells and titrated on Vero cells.

### Virus titration

Classical plaque assay on Vero cells was used to determine viral titers. Briefly, 2.5x10^5^ Vero cells per well were grown in 6-well plates. Ten-fold serial dilutions of the virus stocks were incubated with the cells for 1 h at 37 °C; then, 2 mL of overlay medium (0.375% Low-Melting Point Agarose in DMEM medium supplemented with 5% FBS) was added. Seven days post-infection, cells were fixed with 4% formaldehyde for 4h. Overlay medium was removed and fixed cells were stained with crystal violet solution (0.2% crystal violet, 20% of methanol in PBS) for 20 minutes to reveal the lytic plaques. The well with the number of plaques between 20 and 100 was used to determine the virus titer, which was expressed as plaque forming units per milliliter (PFU/mL).

### Selection of the experimental mouse model

Six-week old immunocompetent Balb/c and C57BL/6 mice (n = 10 per group) were infected via intraperitoneal (i.p.) route with several doses of ROCV or RPMI1640 (mock-infected). Weight loss and lethality rate were measured for up to 21 days post-infection.

### Analysis of the cross-protection against ROCV

A total of 11 groups of mice were employed in our study. Mice groups were denominated as: DENV-1, DENV-2, DENV-3, DENV-4, BSQV, CPCV, ILHV, SLEV, YFV, ZIKV and ROCV, according with the virus used for infection. Six-weeks old C57BL/6 mice were infected intraperitoneally (i.p.) either once or twice with non-lethal doses of different flaviviruses (1x10^6^ PFU) (n = 40 animals per group) or sub-lethal dose of ROCV (276 PFU, n = 60). RPMI inoculated animals were used as control (mock-infected animals). Three weeks after the last infection, humoral immune responses were analyzed. Ten animals per group previously immunized either once or twice were subjected to terminal bleed under ketamine/xylazine anesthesia. Subsequently, 20 animals per group that showed no signs of encephalitis were challenged i.p. with a high dose (2.76x10^7^ PFU) of ROCV; then, the survival rate, clinical score and weight loss were computed for up to 21 days after challenge. Mice were scored from 0 to 5 as described by Miller et al, 2010 [28]. Briefly, 0 corresponds to the signs of disease being absent or the same as in the mock-infected animals, 1 when limp tail or hind limb weakness was present (but not both), 2 when both limp tail and hind limb weakness were present, 3 when partial hind limb paralysis was observed, 4 when complete hind limb paralysis was observed, and 5 for moribund state (euthanized). Mice were monitored thrice daily for signs of disease. Mice with clinical score of 4 were immediately euthanized within 30 minutes by using ketamine/xylazine anesthesia and cervical dislocation.

### Detection of homotypic antibodies

To confirm the production of antibodies against each flavivirus, an enzyme-linked immunosorbent assay (ELISA) was performed. Half of each 96-well plates were coated with supernatant from infected (10^4^−10^5^ PFU of each viruses) C6/36 cells diluted in 0.05 M carbonate-bicarbonate buffer, pH 9.6 (Sigma-Aldrich, USA) at 4°C overnight. Supernatant from uninfected C6/36 cells with carbonate-bicarbonate buffer was added to the other half of the plates to be used for background measurements. Subsequently, the plates were washed three times with phosphate-buffered saline containing 0.05% (v/v) Tween (PBS-T) and were subsequently blocked with 150 μl of blocking solution (PBS-T with 10% (w/v) of non-fat dry milk) for 2 hours at 37°C. The plates were washed again and 50 μl of each immune serum sample (diluted 1:100 in blocking solution) was added in duplicate wells containing the viral antigens and supernatant of uninfected cell culture (n = 10 serum per group). Serum samples of mock-infected animals (n = 10) were used as negative control. After 1 hour of incubation at 37°C, the plates were washed four times with PBS-T and 50 μl of goat anti-mouse IgG conjugated to horseradish peroxidase (HRP) (Sigma-Aldrich, USA) (diluted 1:2000 in blocking solution) was added and incubated for 1 hour at 37°C. Subsequently, the plates were washed five times with PBS-T and 100 μl of 3,3',5,5'-Tetramethylbenzidine (TMB) peroxidase substrate solution (KPL, USA) was added and incubated for 15 minutes at 37°C. Then, 100 μL/well of chlorhydric acid 1 N was added to stop the reaction and absorbance values were measured at 450 nm in a microwell plate reader (Multiskan Ascent 354, USA). The mean absorbance value was calculated for each duplicated serum samples and subtracted by the mean absorbance value of the corresponding background control. The mean absorbance value of the group of animals infected with each virus was compared with the mean absorbance value of the uninfected control group by the *t*-test for significance analysis.

### Plaque reduction neutralization test

To determine the level of neutralizing antibodies, the Plaque Reduction Neutralization Test (PRNT) was used according to PRNT guidelines [29]. Briefly, the serum samples of infected C57BL/6 mice were inactivated at 56°C for 30 minutes and two fold serial dilutions (from 1:5 to 1:10240 in DMEM containing 4% FBS) of the serum samples were incubated with 100 PFU of ROCV at 37°C for 1 hour. Subsequently, each mixture was incubated with Vero cells (2.5x10^5^ Vero cells per well in 6-well plates) for 1 hour at 37°C with gentle agitation every 10 minutes. Overlay medium was added to the infected Vero cells and plates incubated at 37°C with 5% of CO_2_ atmosphere for 3 days. Cells were fixed and stained as described above. The neutralizing antibody titer was defined as the reciprocal of the last serum dilution that inhibited 50% of the lytic plaques formation when compared to cells infected with ROCV incubated with the serum of mock-infected animals and expressed as plaque forming units per well (PFU/well).

### Analysis of evolutionary identity of ROCV between others flaviviruses

The complete E amino acids sequences of 11 flaviviruses used in this study were retrieved from NCBI (reference sequences) and aligned using MEGA 7 program [30]. Divergence was calculated using a *p-*distance model with 1,000 replicates and identities between sequences were calculated.

### Statistical analysis

Gehan-Breslow-Wilcoxon survival curves were analyzed by Log-rank test using Graph Prism 7.0 software. A two-tailed *t* test was used for significance analysis in the T cells responses and homotypic antibody analysis. A *p* value < 0.05 was considered significant.

## Results

### Evaluation of C57BL/6 and Balb/c mice for susceptibility to ROCV infection

**Fig 1:**
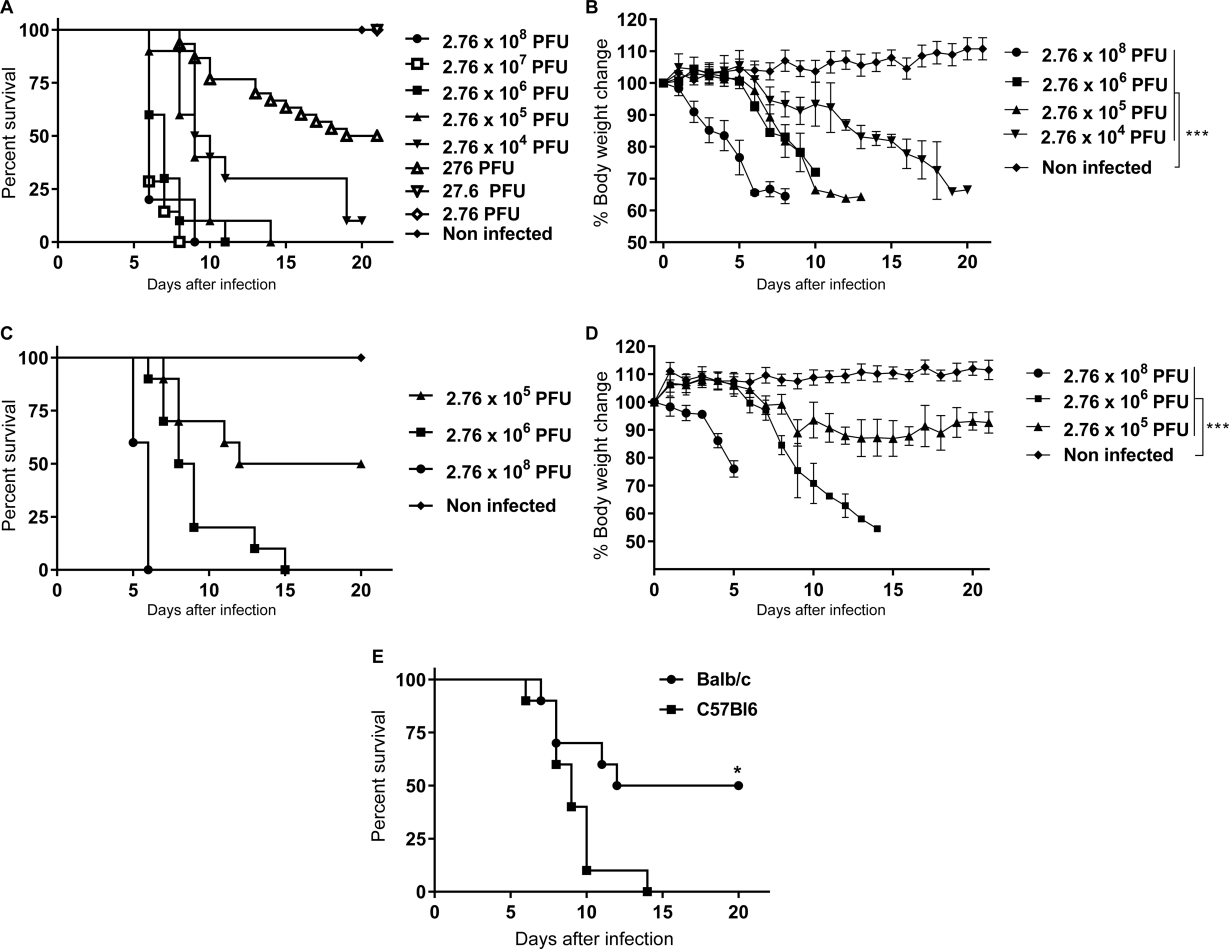
Susceptibility of Balb/c and C57BL/6 mice to ROCV infection. Mice were infected with different doses of ROCV (n = 10 mice per group). Survival rate and body weight loss for C57BL/6 (A and B) and for Balb/c (C and D). Comparison of mortality rate between C57BL/6 and Balb/c (Fig 1E). Survival rate and body weight loss were measured for up to 21 days post-infection. Statistically significant differences in B and D: ****p<0.0001 was determined by one-way ANOVA with Dunnett's multiple comparisons test, in E: *p<0.01 by Log-rank (Mantel-Cox) test.

Initially, the susceptibility of immunocompetent Balb/c and C57BL/6 mice to ROCV infection was determined. Six-week old animals were infected by the i.p. route with different doses of ROCV. Weight loss and lethality rate were measured for up to 21 days (Fig 1). C57BL/6 mice infected with viral doses ranging from 2.76x10^4^ PFU/mouse to 2.76x10^8^ PFU/mouse showed dramatic weight loss and high lethality (90–100%) (Fig 1A and 1B). In Balb/c mice, viral doses of 2.76x10^6^ PFU/mouse and 2.76x10^8^ PFU/mouse were able to induce dramatic weight loss and high mortality rates (100%) (Fig 1C and 1D). However, when the animals were infected with 2.76x10^6^ PFU of ROCV, survival was on average four days longer in Balb/c mice when compared with C57BL/6 mice. In addition, Balb/c mice showed significantly lower mortality rate than C57BL/6 mice when 2.76x10^5^ PFU of ROCV was used for infection (Fig 1E, *p*<0.015). These results demonstrated that C57BL/6 mice were more susceptible to ROCV infection; therefore, these animals were used as the experimental model in this study.

### Analysis of antibody responses induced by infection with different Brazilian flaviviruses

**Fig 2:**
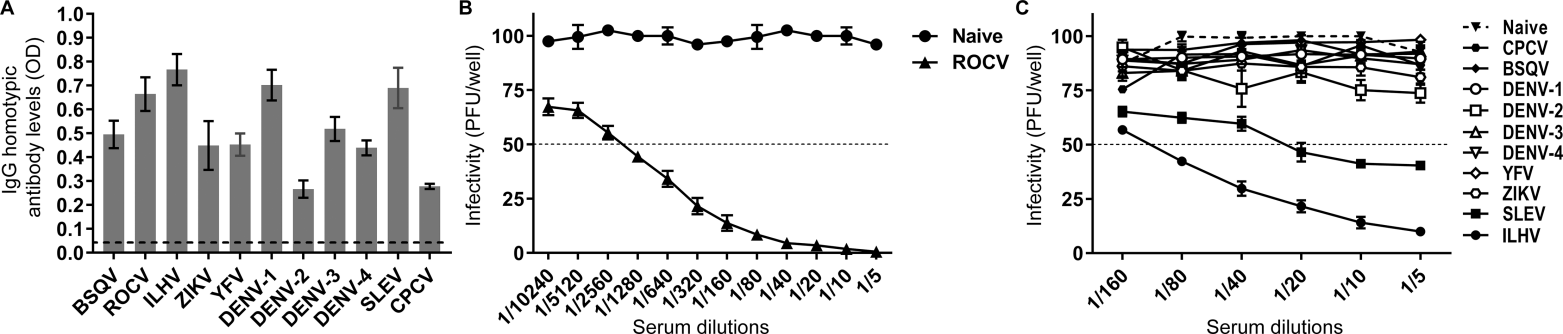
Antibody responses following infection with different flaviviruses. (A) The level of homotypic antibodies (IgG) of a group of 10 animals was compared with the corresponding level of a negative control group (n = 10 mock-infected animals). (B) The level of neutralizing antibodies in C57BL6 mice infected with ROCV (n = 10) and (C) different flaviviruses circulating in Brazil.

To determine homotypic antibody responses elicited against different flaviviruses, C57BL/6 mice were infected twice with 10^6^ PFU/mouse of different Brazilian flaviviruses or a sub-lethal dose (276 PFU/mouse) of ROCV. Sera were collected 2 weeks after the second infections and analyzed by ELISA using respective viral antigens prepared in C6/36 cells. The results showed that all infections produced homotypic antibody responses (Fig 2A). Serum samples were also analyzed for neutralizing antibodies against ROCV by the virus-neutralization (PRNT) assay (Fig 2B and 2C). Although the highest level of neutralizing antibodies (PRNT_50_ titre ~1:1280) was found in animals infected with a sub-lethal dose of ROCV (Fig 2B), the animals that were previously infected with ILHV and SLEV but not with the other flaviviruses, also elicited cross-neutralizing antibody responses against ROCV. PRNT_50_ titers were ~1:80 and ~1:20 for ILHV and SLEV, respectively (Fig 2C).

### Prior infection of mice with ILHV and SLEV, but not with other Brazilian flaviviruses, induces cross-protection against lethal ROCV challenge

**Fig 3:**
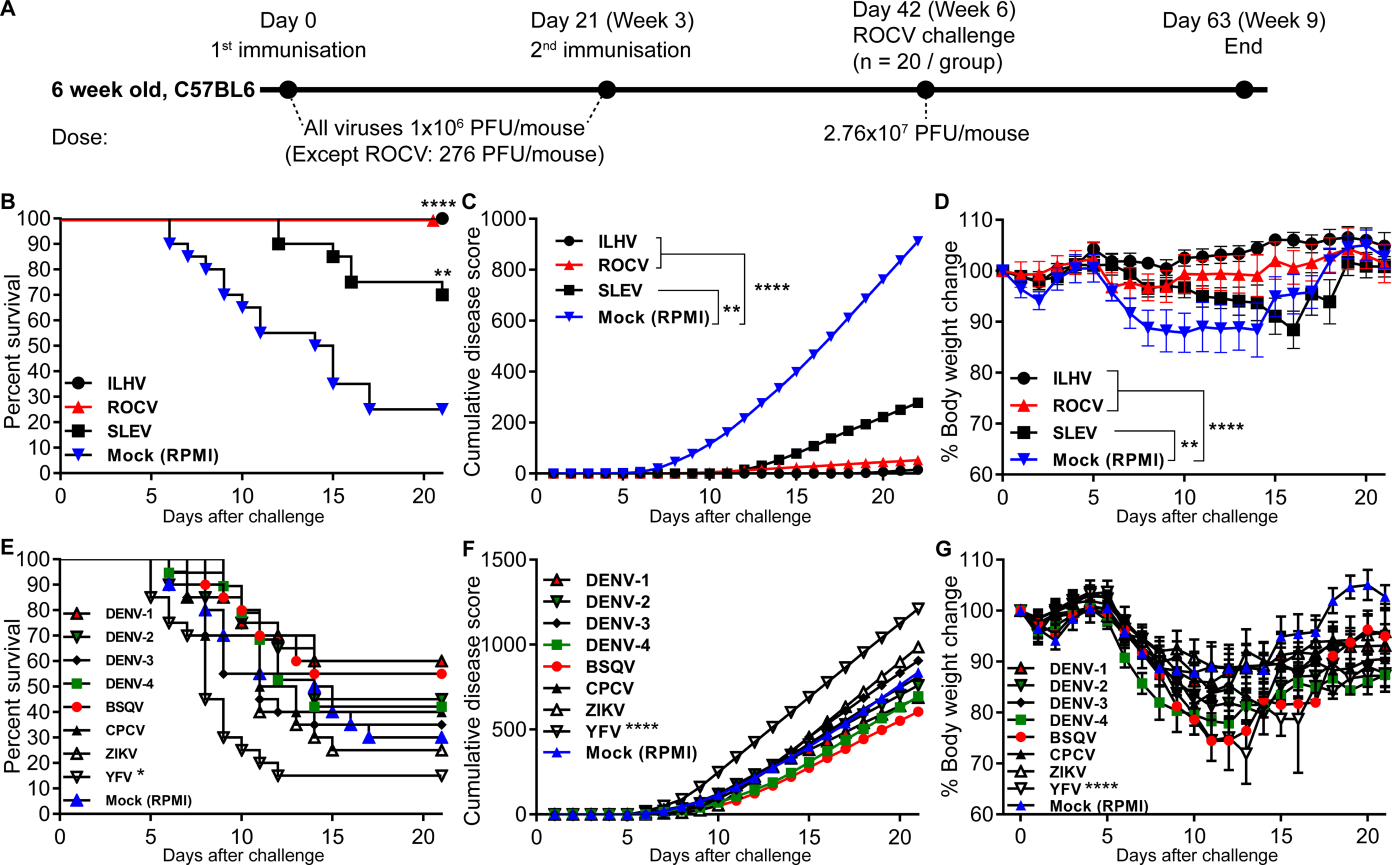
Evaluation of cross-protective immunity against ROCV after prior infections with different flaviviruses. Immunization and challenge regime (A). Mice (n = 40 per group) were infected twice with the different flaviviruses known to circulate in Brazil, and were then challenged (n = 20 per group) with 2.76x10^7^ PFU of ROCV and survival rates (B and E), clinical scores (C and F) and body weight loss (D and G) were determined up to 21 days post-infection. Statistically significant differences in B and E: *p<0.01, **p<0.001 and ****p<0.0001 was determined by Log-rank (Mantel-Cox) test. In F: ****p<0.0001 was analyzed by a student t-test. In D-G *p<0.01, **p<0.001 and ****p<0.0001 was determined by One-way ANOVA with Dunnett's multiple comparisons test. All statistically significant differences were with group control (Mock).

We demonstrated that a dose of ROCV at 276 PFU/animal resulted in 50% survival (Fig 1A), and was used as the immunization dose as a control for a homotypic response in the survivors. To assess cross-protection by other flaviviruses, we performed an immunization/challenge regime as detailed in Fig 3A. Animals immunized with BSQV, DENV (1 to 4), YFV and ZIKV showed no disease symptoms—and resulted in 100% survival (S1 Fig). While animals immunized with ILHV, CPCV and SLEV displayed 10% mortality (S1 Fig)—the remaining 90% survivors did not show any symptoms and were used for ROCV challenge.

Twenty survivors per group from the immunization showing no disease symptoms were used for challenge with a lethal dose (2.76x10^7^ PFU/mouse) of ROCV. Only groups of animals previously infected twice with ILHV, SLEV, or infected with a sub-lethal dose of ROCV, showed higher survival rates (Fig 3B), lower clinical scores (Fig 3C) and absence of weight loss (Fig 3D), when compared to the group not previously infected with any virus. Prior infections with ILHV virus as well as prior infection with a sub-lethal dose of ROCV were most efficient in protecting mice, with 100% of mice surviving ROCV challenge (Fig 3B).

Prior infections with other Brazilian flaviviruses (DENV-1, DENV-2, DENV-3, DENV-4, BSQV, CPCV and ZIKV) produced variable effects on the following challenge with a lethal dose of ROCV, ranging from mild protection to enhancement of infection, although these differences in protective/enhancement effects did not show any statistical significance compared to mock (Fig 3E–3G). Interestingly, rather than providing protection against ROCV infection, YFV-immunized animals showed significantly higher mortality, disease scores and body weight changes compared to mock (Fig 3E–3G).

**Fig 4:**
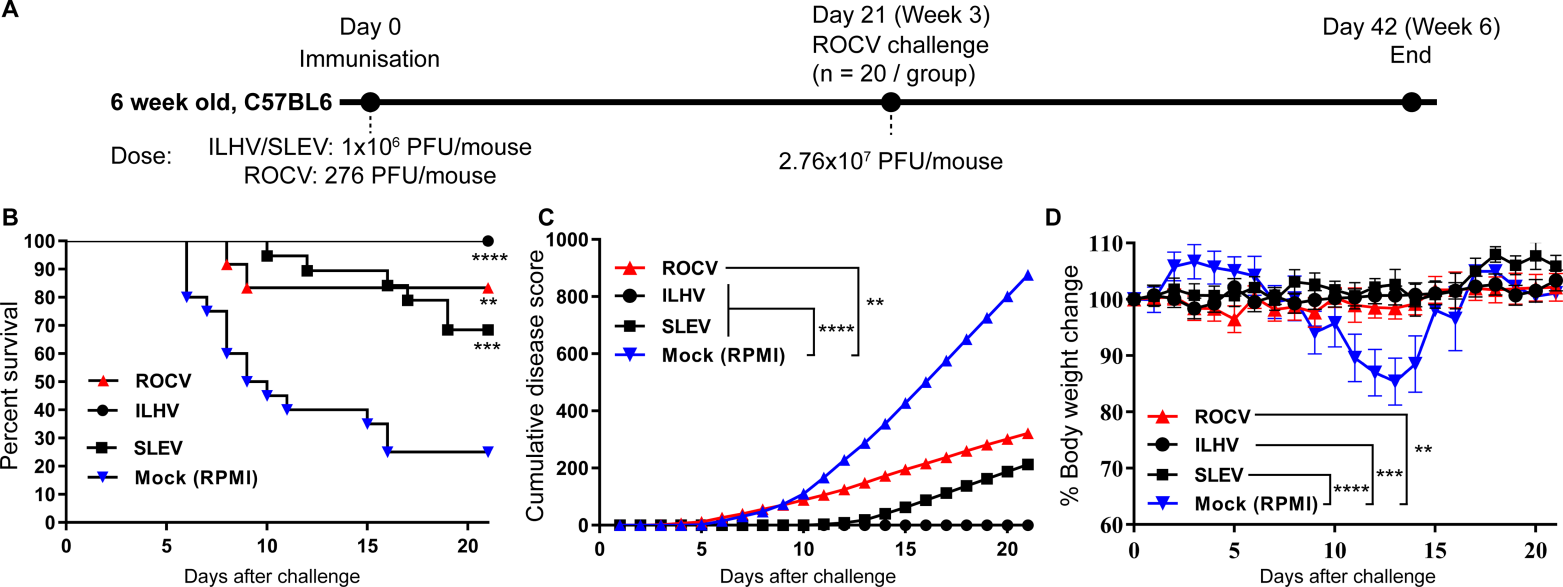
Evaluation of *in vivo* cross-protective immunity against ROCV after a single infection with ILHV and SLEV. Immunization and challenge regime (A). Mice (n = 20 per group) were infected once with ILHV or SLEV or ROCV and were then challenged with 2.76x10^7^ PFU of ROCV. Survival rates (B), clinical scores (C) and body weight loss (D) were then determined up to 21 days post-infection. Statistically significant differences in A: **p<0.001, ***p<0.0001 and ****p<0.0001 was determined by Log-rank (Mantel-Cox) test. In B-C: ****p<0.0001 was determined by One-way ANOVA with Dunnett's multiple comparisons test.

To determine if a single prior infection was sufficient to confer protection, mice that were also infected once with ILHV or SLEV or a sub-lethal dose of ROCV, were then challenged with a lethal dose of ROCV. A single ILHV infection was most effective in protecting mice against subsequent lethal ROCV challenge (100% survival), followed by a sub-lethal dose of ROCV (80% survival), and infection with SLEV (68% survival) (Fig 4).

These results clearly demonstrate that prior infection with ILHV or SLEV confers cross-protection against subsequent ROCV infection in C57BL/6 mice.

## Discussion

In the present study, we show that C57BL/6 mice infected with both ILHV and SLEV elicited cross-neutralizing antibodies against ROCV and that prior infection of mice with ILHV provided complete protection against subsequent lethal challenge with ROCV. While prior infection with SLEV significantly reduced mortality following subsequent lethal ROCV challenge. Interestingly, serologic analysis of sera from ROCV-infected people in 1975 outbreak showed strong cross-reactivity with ILHV and SLEV viruses by complement fixation test, but only anti-SLEV and not anti-ILHV sera was able to neutralize ROCV infection in cell culture [1]. The extent of cross-neutralization among flavivirus-induced antibodies is believed largely to correlate with the amino acid sequence homology of the viral surface E glycoprotein [31]. Phylogenetic analysis illustrated that ROCV is more closely related to ILHV and SLEV than to other members of the *Flavivirus* genus [10, 32]. The high amino acid sequence similarity in the E protein between these viruses (Table 1) is likely responsible for the cross-protection found in the present study. It is important to note that although CPCV and SLEV share a 65% amino acid sequence identity with the E protein of ROCV, amino acid identity does not take into account differences in conformational/linear epitope availability. Furthermore, quaternary epitopes between E/E or E/prM cannot be represented just by amino acid identity values. We can only speculate that CPCV is missing an important epitope for cross-protection.

**Table 1:** Amino acid identity between the E proteins of Brazilian flaviviruses used in this study.

Virus	1	2	3	4	5	6	7	8	9	10
**(1) ROCV**										
**(2) ILHV**	78.8									
**(3) SLEV**	65.5	67.4								
**(4) CPCV**	65.1	63.8	69.9							
**(5) BSQV**	53.2	54.3	56.5	53.8						
**(6) DENV-1**	48.2	49.9	50.7	51.6	49.3					
**(7) DENV-2**	47.4	50.1	49.3	48.0	48.9	69.2				
**(8) DENV-3**	49.5	49.3	49.5	49.7	48.4	78.4	68.2			
**(9) DENV-4**	47.0	46.2	48.2	50.5	52.4	63.8	63.6	63.4		
**(10) ZIKV**	55.3	55.5	55.5	54.9	54.7	58.4	54.7	59.3	57.0	
**(11) YFV**	44.9	44.5	45.7	43.9	42.4	42.8	45.3	41.8	40.7	43.0

The analysis was conducted in MEGA7 program. The identities (%) of ROCV between different Brazilian flaviviruses are shown. GenBank Protein accession no.: ROCV (AAV34158), ILHV (AAV34155), SLEV (YP_001008348), CPCV (CRI72595), BSQV (AAV34152), DENV-1 (BAM08265), DENV-2 (AAC59275), DENV-3 (AAA99437), DENV-4 (AAA42964), ZIKV (YP_009430300), YFV (CAA27332).

Interestingly, we observed that prior immunity against YFV enhanced mortality and disease score upon challenge with ROCV, compared to the mock group—suggesting an effect by antibody-dependent enhancement (ADE). Although further experiments are necessary to prove the ADE hypothesis, we highlight the potential complications that could arise if a ROCV outbreak were to occur in a population vaccinated with the YFV vaccine strain (used in this study)—especially with fractional doses, which could generate sub-optimal antibody responses, such as is currently occurring in Brazil.

Although no more cases of encephalitis attributed to ROCV was reported in Brazil after the 1976 outbreak, serological surveys for flaviviruses in human and animals have suggested that unreported and therefore likely mild infections with ROCV still occur in several regions of Brazil, and in some cases, as co-infections with other flaviviruses, including ILHV and SLEV [6, 8, 9, 33, 34]. ROCV has not been detected outside Brazil, while ILHV and SLEV are widely distributed among humans and animals in the Americas [35–41]. These epidemiological observations together with our experimental data in mice support the hypothesis that the immunity induced by ILHV and SLEV is cross-protective against ROCV infection. This may lead to the apparent disease-free circulation of ROCV in Brazil identified by serological surveys. However, the mis-diagnosis of ROCV infections by serological surveys due to the cross-reactivity with other flaviviruses cannot be completely excluded in some cases, thus justifying a need for developing other more accurate diagnostic assays to define whether ROCV is in fact circulating in Brazil. New high throughput next-generation sequencing methods are able to detect known and unknown viruses. These methods have been shown to be useful for the identification of other flaviviruses causing encephalitis like WNV and SLEV in the cerebrospinal fluid of patients [42, 43], but they are still too expensive to be used in routine surveillance systems [44]. A more economical alternative to the next-generation sequencing methods is microarray technology, which has the potential for screening thousands of viruses in a single assay. In that sense, we have recently developed a microarray platform for the screening of 416 viruses (arboviruses and viruses transmitted by small mammals) in all kinds of clinical samples [45], which could be used for the surveillance of arboviruses, including ROCV.

In summary, we have shown that pre-existing infection with ILHV or SLEV is able to cross-protect against subsequent infection with ROCV in mice, supporting the view that pre-existing immunity induced in humans by these endemic Brazilian flaviviruses may attenuate subsequent ROCV infections and thus explain the lack of reports of severe ROCV infections.

## Supporting information

S1 Fig Evaluation of survival rate after immunization with different flaviviruses. Mice (n = 40 per group) were infected once with the different flaviviruses known to circulate in Brazil. Groups (DENV-1, DENV-2, DENV-3, DENV-4, BSQV, CPCV, ILHV, SLEV, YFV and ZIKV) were denominated according with the virus used for infection.
(TIF)
