# Supplementary figures and images for: Ilheus and Saint Louis encephalitis viruses elicit cross-protection against a lethal Rocio virus challenge in mice
Source: PLoS One. 2018 Jun 13;13(6):e0199071. doi: 10.1371/journal.pone.0199071 (PMC5999289; doi:10.1371/journal.pone.0199071)

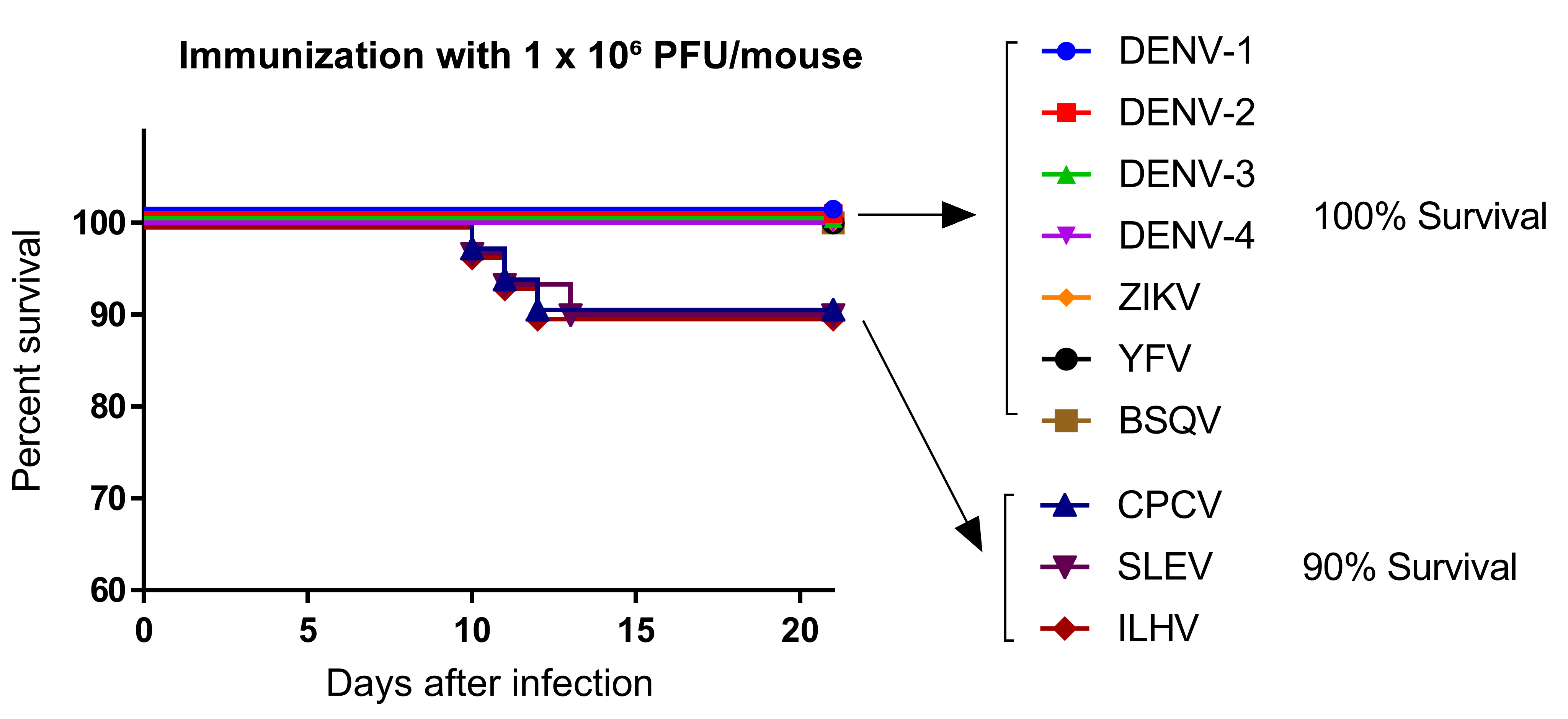

Supplement: S1 Fig — Mice (n = 40 per group) were infected once with the different flaviviruses known to circulate in Brazil. Groups (DENV-1, DENV-2, DENV-3, DENV-4, BSQV, CPCV, ILHV, SLEV, YFV and ZIKV) were denominated according with the virus used for infection. (TIF) [file pone.0199071.s001.tif]
